# Provider-Engaged Development of a Sexual Dysfunction Screening Approach for Adolescents and Young Adult Childhood Cancer Survivors: Iterative Co-Design Study

**DOI:** 10.2196/85905

**Published:** 2026-04-08

**Authors:** Jenna Demedis, Julia Reedy, Eric J Chow, Pamela N Peterson, Brooke Dorsey, Christina R Studts

**Affiliations:** 1 Department of Pediatrics, University of Colorado School of Medicine, 13123 E. 16th Ave B115, Aurora, CO, 80045, United States, 1 720-777-0188; 2 Center for Cancer and Blood Disorders, Children's Hospital Colorado, Aurora, CO, United States; 3 Adult and Child Center for Outcomes Research and Delivery Science (ACCORDS), University of Colorado School of Medicine, Aurora, CO, United States; 4 Fred Hutchinson Cancer Center, Clinical Research and Public Health Sciences Divisions, Seattle, WA, United States; 5 Department of Internal Medicine, University of Colorado School of Medicine, Aurora, CO, United States; 6 Denver Health Medical Center, Denver, CO, United States; 7 Department of Family Medicine, University of Colorado School of Medicine, Aurora, CO, United States

**Keywords:** pediatric cancer, sexual dysfunction, health services research, adolescent and young adult cancer survivors, dissemination and implementation science, user-centered design, co-design

## Abstract

**Background:**

Sexual dysfunction (SD) is common among childhood cancer survivors, affecting approximately 20% to 50% of patients. National guidelines recommend discussions about sexuality throughout cancer care, and prior work demonstrates patient interest in SD conversations. Despite its prevalence and importance, SD is widely underrecognized and undertreated, creating gaps in comprehensive whole-person care.

**Objective:**

Our research aims to collaborate with provider partners to co-design an SD screening intervention prototype for implementation in a clinical oncology setting. This study outlines the co-design process to serve as a case study, highlighting challenges and strategies to achieve a consensus-driven intervention and implementation plan.

**Methods:**

We engaged pediatric cancer providers in a series of co-design sessions at a National Cancer Institute–designated cancer center within an academic children’s hospital. For each co-design session, the research team created a template outlining considerations from formative work (eg, patient privacy) and key decisions to be made (eg, screening modality). Co-design session moderators facilitated discussion, guiding participants toward a consensus decision for each intervention component. A final process mapping session reviewed and outlined the entire SD prototype. We conducted a rapid qualitative analysis, compiling a templated summary synthesizing and organizing findings by discussion topic and decision point. Based on co-design discussions, the research team compiled a menu of options outlining key thematic findings, core screening intervention functions, and intervention form options to allow for future expansion and tailoring of the SD prototype.

**Results:**

Six provider participants, including attending physicians, advanced practice providers, and registered nurses representing multiple oncology subspecialty groups, engaged in a series of 5 co-design sessions. Participants assessed specific intervention component options, reached consensus on component decisions, and determined an intervention and implementation workflow for each. Throughout, providers needed to ensure workflows aligned with patient and provider priorities from foundational work and to ensure design feasibility, acceptability, and appropriateness. Key intervention and implementation decisions included target population, screening frequency, screening modality and workflow, management of screening results, clinic reminders and cues, and provider education and training. With several decisions being interconnected, there was often a cascade effect in which one decision influenced or limited future decisions and, in some cases, required revisiting prior decisions to ensure cohesive alignment into a single prototype. Co-design session moderators used several strategies (eg, reminders, redirection, providing information on feasibility, etc) to facilitate decision-making and implementation strategy selection.

**Conclusions:**

Engaging provider partners in co-design sessions allowed for the collaborative development of a preliminary SD screening approach for adolescents and young adults with and surviving cancer. The dynamic co-design process and moderator strategies ensured that intervention and implementation decisions reflected the patient and provider priorities identified in prior work. Future work will test, adapt, and refine the prototype SD screening approach prior to effectiveness testing and eventual dissemination.

## Introduction

### Background

Childhood cancer survivors are at risk for sexual dysfunction (SD) as a result of their cancer history and treatment [1-4]. Unfortunately, while SD affects between 20% and 50% of childhood cancer survivors, it is underrecognized and undertreated, leaving a gap in whole-person health care for these patients [1,2]. This unmet need exists despite national recommendations that sexual health, including sexual function, be addressed by oncology providers throughout the cancer care continuum [5-7]. Prior studies have identified numerous patient-level and provider-level barriers to SD communication, such as patient or provider discomfort and privacy concerns, which may be heightened in adolescent and young adult childhood cancer survivors [8-11]. Our prior research suggests that some of these barriers may be addressed through the use of standardized, patient-centered SD screening [9,11,12].

Unmet SD needs among adolescent and young adult childhood cancer survivors could be identified and addressed in pediatric oncology clinics, where patients are followed throughout and after treatment. Standardized, patient-centered SD screening could serve as the first step in ensuring appropriate sexual health care for this population. However, the routine use of patient-reported outcome measures is not common practice in pediatric oncology clinics, and barriers to implementation exist [13-15]. Additionally, SD in adolescent and young adult childhood cancer survivors can be a sensitive topic for both patients and clinicians unaccustomed to discussing this topic in oncology care [8,10,11]. These challenges are among several foreseeable barriers to the implementation of SD screening in this setting related to privacy, workflow, patient and provider buy-in, and more [9].

### Study Frameworks: Context, Outcomes, and Process

To develop an implementable patient-centered SD screening approach, we applied implementation science and user-centered design principles in an active, collaborative, and iterative approach to intervention design and implementation [16-18]. Through our prior and current work, this process leveraged considerations of context (guided by the CFIR [Consolidated Framework for Implementation Research] [19]) and engagement of oncology clinic partners to co-design an SD screening approach. We aimed to develop an approach that would fit within the contexts of pediatric oncology clinics, with future goals of achieving high Reach, Effectiveness, Adoption, Implementation, and Maintenance (RE-AIM) [20,21]. The RE-AIM and CFIR frameworks have been used successfully in many fields to systematically assess the robustness of interventions across settings and patient subgroups, with attention to the potential for scaling up to additional settings.

To support future dissemination and implementation of SD screening in pediatric oncology care, this study sought to develop a flexible intervention and implementation plan that can be tailored to a variety of contexts and practice settings. To do so, we adopted the implementation science–grounded concepts of “functions” and “forms” of an intervention: functions being the fundamental purpose or desired effect of an intervention or implementation strategy and forms being the intervention or strategy details, components, and activities selected to achieve specified functions. To achieve functions, forms may vary based on contextual factors influencing implementation [22,23].

Across this multiphase project, we followed the Wisconsin Interventions for Team Science Framework, beginning with a discover phase followed by an iterative design-build-test phase for the prototype SD screening approach [16]. In the discover phase, we first engaged patients and then providers through qualitative interviews, guided by the CFIR, to elicit perspectives on SD screening in the pediatric oncology setting, including potential barriers and facilitators that might impact implementation success as defined by RE-AIM outcomes [9,11,12]. These initial studies verified the need for a standardized SD screening intervention, identified the key functions of an SD screening approach, and highlighted potential forms for intervention and implementation design [9,11,12,24]. Both patient and provider participants found the National Institutes of Health Patient-Reported Outcomes Measurement Information System (PROMIS) Sexual Function and Satisfaction v2.0 Brief v2.0 (SexFS Brief) tool to be acceptable for this population and favored its use in facilitating SD conversations [9,11,12,25-27]. This tool is available in versions for male and female patients and is validated in adult cancer populations [25-27]. Subsequently, the design phase of the SD screening approach development process used collaborative, iterative co-design sessions with a group of pediatric oncology providers.

We frame this paper as a case study of the development of a consensus-driven intervention and implementation plan for an SD screening approach using the SexFS Brief in pediatric oncology clinics. The methods and results of this paper describe our process for engaging prospective users to determine the forms of the SD screening approach comprising the resulting prototype. More contentious or difficult decisions are highlighted, accompanied by descriptions of strategies for arriving at consensus in these scenarios. We also provide details about the specific design considerations and decisions for our SD screening approach, which can be used in future research investigating its effectiveness and potential for wide-scale dissemination and implementation.

## Methods

### Study Design

The intervention and implementation development process used a series of co-design sessions [28-32] with a multidisciplinary group of pediatric cancer providers (physicians, advanced practice providers, and nurses) to develop an SD screening approach using the SexFS Brief in pediatric oncology clinics. Co-design session content was informed by the prior discover phase, in which providers and patients shared their preferences and recommendations for SD screening in qualitative interviews [11,12]. With the goal of developing a prototype SD screening approach through collaborative decision-making, as opposed to idea generation or brainstorming and reflecting, we engaged the same group of provider participants throughout the iterative design phase, following user-centered design principles [17]. The results of this process (ie, functions and forms of the SD screening approach as well as themes defining the decision points during the development process) will be used in the build and test phases, aiming to refine and pilot test the implementation and effectiveness of the SD screening approach.

### Ethical Considerations

All study procedures were reviewed and approved by the Colorado Multiple Institutional Review Board (20‐1679). Reporting follows the StaRI (Standards for Reporting Implementation Studies) guidelines, with the co-design process considered the implementation strategy [33]. All participants provided consent for their participation in this research at the beginning of the first co-design session. Upon completion of the 5 planned sessions, each participant received a US $100 gift card.

### Study Site

This study was conducted at a National Cancer Institute-designated cancer center within a quaternary academic children’s hospital in the western United States. The children’s hospital is a referral center with approximately 27 oncology physicians treating over 300 patients with cancer each year, drawing from an 8-state catchment region. The patient population is largely non-Hispanic White, with approximately 45% insured by Medicaid.

### Sample: Multidisciplinary Design Team

Participants for the design phase were purposively recruited from provider interviews conducted in the discover phase (N=25), exploring perspectives on the use of the SexFS Brief in pediatric oncology clinics [9]. Eligible individuals were the 25 discover phase pediatric oncology medical provider participants serving in any of the following roles: faculty physician, fellow physician, advanced practice provider, registered nurse serving as a nurse care coordinator, or medical assistant (MA). There were no exclusion criteria. Co-design sessions require substantial participant engagement and rely on thoughtful in-depth discussion; additionally, our design phase participants were expected to attend several sequential co-design sessions and ultimately arrive at consensus decisions. Therefore, consistent with other studies involving medical providers in the co-design of implementation strategies [32,34-36], we sought to engage a smaller total sample size of 6 to 8 highly engaged participants. Recruitment was completed on a rolling basis with targeted outreach to achieve variation by provider role and clinical team (ie, leukemia/lymphoma, central nervous system tumors, or solid tumor). In addition to the desired variation in clinical roles, the research team considered the depth of discover phase interview responses in determining the order in which to recruit eligible participants, aiming to balance variation in roles with demonstrated capacity to share perspectives. A total of 10 participants were approached, 9 expressed interest in participation, and 6 were selected for the final sample based on the alignment of schedules and ability to commit to all or nearly all co-design sessions. The final group of participants (n=6) who committed to attending planned co-design sessions included all provider roles except for MA and represented each clinical team.

### Co-Design Session Procedures

#### Preparation

The study team (JD, JR, CRS) developed a template for each of the 5 planned sessions. The templates, informed by prior interviews with patients and providers, identified key intervention design and implementation decisions to be reviewed and discussed [9]. For each session, the data from previous patient and provider qualitative interviews were integrated to develop (1) a list of key decisions to be made; (2) a preliminary list of options and recommendations for each of these decisions, generated by the research team based on the prior interviews; and (3) overarching key considerations to be prioritized in decision-making (such as privacy and workflow). For example, the first co-design session focused on determining the target population and the modality for screening delivery. The key decisions regarding target population and modality, options and recommendations for these intervention and implementation components, and overarching considerations to prioritize during decision-making were summarized and displayed during the co-design session. Topics of each co-design session are provided in the *Results* section and Table 1.

**Table 1.** Outline of co-design session content for the development of a sexual dysfunction (SD) screening approach^a^.

Focus	Content
Session 1	
Background	Overview of study purpose and process mapping Overarching design considerations
Intervention	Target patient population Modality for screening delivery
Session 2	
Intervention	Session 1 review Screening workflow/timing Results management plan (provider responsibilities) Location of sexual dysfunction screening EHR^b^-specific privacy issues
Session 3	
Intervention	Session 2 review Follow-up on unanswered logistical questions from session 2 (EHR capabilities) Revisiting results management plan Frequency of screening Screening results storage
Implementation	Introduction of screening to patients or families Patient-centeredness opportunities (tailoring, choice) Workflow or capacity constraints, scheduling opportunities
Session 4	
Implementation	Session 3 review Accountability/sustainability (reminders, guidelines, feedback) Role of clinical champion, leadership team Provider buy-in Resource needs Provider training Patient education
Session 5	
Implementation	Session 4 review Updating or revisiting reminders, feedback, and patient education Overview of complete prototype Process mapping

a Outline of 5 co-design sessions which took place from December 2021 to February 2022. The table includes the focus of each session and the content and key decision points discussed in each. Each session included a review (session 1 review focused on overarching design considerations from prior findings) to ensure alignment with previous findings to facilitate creation of a single, streamlined prototype.

b EHR: electronic health record.

#### Session Structure

Five iterative co-design sessions were conducted via the Zoom video-conferencing platform with end-to-end encryption. Sessions were 60 minutes in length and were digitally recorded with participants’ permission. Each session was moderated by JD, the principal investigator and a colleague of the participants. Sessions were observed and comoderated by JR, a trained qualitative analyst who collected detailed field notes and created summaries during and immediately after each session, grouping notes by topical discussion. In the event of a participant’s absence, a member of the research team independently met with the participant to review the session summary and elicit that participant’s insights.

Each co-design session focused on one or more topics informing the SD screening approach design or implementation. The first co-design session included an overview of the overarching goal of the project: to develop a patient-centered, feasible approach to standardized screening for SD in pediatric oncology patients, using key user input. The goal of the co-design process itself was also outlined, reminding participants that input was welcome across all roles and that they should aim to reach consensus about various decisions for the intervention and implementation design. Subsequent sessions began with a review of the prior session’s results, serving both as a reminder and as member checking [37]. Topics were introduced along with relevant findings from interviews with providers and patients from the discover phase [9,11,12], with examples of how specific considerations identified in that phase could impact design. Each topic was then opened for discussion and concluded with consensus decision-making for a particular aspect of the SD screening approach. If consensus could not be reached or additional information was needed, the topic was revisited at a subsequent session. If a consensus was not reached after a topic was discussed in more than 2 sessions, the majority opinion was used for decision-making.

The final co-design session involved process mapping to finalize the entirety of the prototype SD screening approach. This session was also used to anticipate potential challenges, resource needs, and promising strategies for facilitating implementation success.

### Data Collection

Demographic and professional characteristics were collected from participating providers through a brief background questionnaire during the discover phase. Audio recordings and field notes for each session served as qualitative data for analysis.

### Analysis

This study used rapid qualitative analysis with a deductive-inductive approach, identifying key themes emerging from co-design sessions. The research team members who were engaged in the co-design sessions and qualitative analyses (JD and JR) practiced reflexivity throughout, including reflective discussions during post-session debriefs and collaborative analysis meetings. As biases of research team members were identified and noted, their potential effects on co-design facilitation and qualitative analyses were discussed with a team member (CS) who was not present at co-design sessions, was less immersed in data collection and analysis, and did not have clinical or research relationships with participants. Through this approach, alternative interpretations of the process and data through an implementation science lens were included to minimize the effects of bias on the research process. In addition, when the research team agreed internally about the potential feasibility of co-design decisions made by participants, strategies such as gathering and objectively presenting feasibility data were used to revisit decisions and ensure consideration from multiple perspectives (moderator strategies highlighted in the *Results* section).

After each co-design session, the co-moderator (JR) synthesized the data from field notes and audio recordings to develop a triangulated summary of the session. Summaries detailed the SD screening approach decision points discussed, including barriers, facilitators, potential solutions, variations in opinions expressed, and any consensus decisions that were made. The summary also included an assessment of the quality of the discussion (eg, participant insight, participation level, rapport, problems, line of questioning). After each session summary was developed, the research team (JD, JR, CRS) met to assess the accuracy and thoroughness of the session summary, referring to field notes and the audio recording as needed. This deductive-inductive approach allowed for organization by co-design discussion topics or decision points and the identification of expected themes based on the CFIR and on interviews conducted in the earlier discovery phase (eg, clinician discomfort discussing SD, relative priority of SD in the pediatric oncology setting) as well as emergent themes. The products of these analytic meetings were revised session summaries for each co-design session, organized by design decision. Session summaries were used to identify decision points characterized by little or no consensus among multidisciplinary design team members and those characterized by high levels of consensus. The results of each session’s rapid qualitative analysis were integrated into the information provided during the next co-design session as a form of member checking, in addition to any clarifications or additional information requested by providers (eg, information about electronic health record [EHR] capability, details about privacy or confidentiality regulations).

Following the completion of the co-design sessions, the research team completed a modified function and form matrix [24], mapping (1) the key themes of discover phase patient and provider interviews, (2) the core functions of the co-designed SD screening approach and strategies to support its implementation, and (3) the forms (options for intervention content and delivery to meet the defined core functions of the screening approach) recommended in the co-design process. Implementation strategies were also mapped to the Expert Recommendations for Implementing Change compilation of 73 established implementation strategies [22,38]. The product of this part of the design phase was the prototype SD screening approach.

## Results

### Overview

The 4 co-design sessions and the process mapping session took place between December 2021 and February 2022. The 6 co-design provider participants are described in Table 2.

**Table 2.** Co-design provider participant characteristics.

Characteristic	Participants, n (%)
Sex	
Female	6 (100)
Race or ethnicity	
White or non-Hispanic	5 (83)
Hispanic	1 (17)
Provider type	
Advanced practice provider	2 (33)
Attending physician	1 (17)
Registered nurse	3 (50)
Oncology subspecialty group	
Leukemia or lymphoma	3 (50)
CNS^a^ tumors	1 (17)
Solid tumor (excluding CNS)	2 (33)

a CNS: central nervous system.

Four co-design sessions were attended by five participants, and one session was attended by four participants. The moderator (JD) met individually with absent participants following missed sessions to update them on the discussion and elicit their input. The content of each session is shown in Table 1.

The findings were organized into 2 major overarching categories consistent with the goals of the co-design process: intervention design and implementation strategy selection. Within intervention design, discussions focused on 7 decision components: target population for screening, introduction of the SD topic and the need for screening of patients, screening modality, workflow, timing and frequency, patient-specific or population-specific tailoring, and clinical management of SD screening results (Table 3).

**Table 3.** Sexual dysfunction screening intervention design topics, considerations, and decisions^a^.

Components	Design considerations	Site-specific plan
Target population		
Age or development	Patient comfort Acceptability Laws or regulations	Age >15 years
Patient status	Acceptability	All patients
Patient choice versus “mandatory”	Patient comfort Reach	Choice to “opt out” of screening Questions can be skipped
Introduction or patient education		
Initial introduction	Interprovider variability Workflow Patient comfort	Initial primary team education Integrate into patient education Integrate in fertility consultation
Re-introduction	Patient comfort Feasibility and workflow	Verbally explain at administration Explanation written on tool
Screening modality		
Written versus verbal	Patient or provider comfort Workflow Future access	Written
Paper versus electronic	Patient comfort Confidentiality Workflow Future access	Electronic
Electronic modality	Fidelity Confidentiality or privacy Workflow Future access	EHR^b^: tablet in clinic with automatic trigger when screening is due
Workflow		
Screening timing	Patient comfort Fidelity or reach Workflow integration	During regular visit
Physical location	Patient comfort Privacy Workflow constraints	Exam room
Workflow for in-clinic	Patient comfort Privacy Workflow constraints	Room patient separately from parent
Screening timing		
Frequency	Flexibility or window of time Patient comfort Feasibility	Maximum of every 3 months (flexible if patient in clinic less often) EHR-based automatic triggers
Initiation of screening	Patient comfort Relative priority	Initiate 1 month after diagnosis
Tailoring delivery		
Language	Patient comfort Validity of screening Feasibility (timing)	Verbal screening unless translation becomes available
Cognitive impairment	Patient comfort Feasibility (time)	Provider appropriateness discussions If appropriate, verbal screening
Sexual function management		
Which provider	Knowledge and comfort Standardization Capacity and workflow Patient comfort or rapport	Dedicated sexual health team with representatives across clinical teams (liquid tumor, solid tumor, neuro-oncology, survivorship)
Timing of SF^c^ discussion	Feasibility constraints Risk loss-to-follow-up	Same visit with telehealth as back-up Consider adding time to appointments
Results storage (EHR)	Confidentiality Workflow (future access)	Results in flowsheets Documentation as minor consent note No limits on provider access

a This table highlights key intervention topics, considerations, and decisions made throughout the co-design process for the development of a sexual dysfunction screening intervention. Organized by topic (eg, target population), we present intervention components requiring design decisions, design considerations for both providers delivering as well as patients receiving the intervention, and the site-specific plan determined by group consensus.

b EHR: electronic health record.

c SF: sexual function.

Within the implementation strategy selection, discussions addressed ways to increase clinician acceptability, buy-in, and adoption of the SD screening approach, including reminders, provider education, resources, and ongoing involvement in design, implementation, and adaptation (Table 4).

**Table 4.** Sexual dysfunction screening approach implementation strategy topics, options, and decisions^a^.

Component (implementation target)			
Subcomponent	Implementation options	Implementation plan	Rationale for initial plan
Reminders (reach, adoption, implementation/fidelity, sustainability)			
Written	EHR^b^-based automatic triggers Dedicated team monitors when due EHR-based (BPAs^c^, blue sticky notes) Paper-based or existing clinic documents	EHR-based questionnaire automatically triggers	Feasibility Integration into workflow Standardization and sustainability (not reliant on team member initiation) Minimal notification fatigue
Verbal	Clinical champions	Project lead Dedicated team	Someone leading implementation efforts Point person for questions or concerns Verbal reminders beneficial
Auditing and feedback	Regular updates on screening rates Notification of missed screening	No auditing	Feasibility Notification fatigue
Provider education (effectiveness, adoption, implementation [acceptability, appropriateness])			
Modality	Emails Educational materials Presentations and meetings Online modules	Meetings with faculty or nurses Written resources Online modules	Multimodal education for increased exposure and variable learning styles
Content	In depth versus overview	Tailored to role Emphasize patient desire for SF^d^ screening Explain need	Tailoring education to the role is feasible and acceptable Different roles have different needs and backgrounds
Who	Only dedicated team members All providers	All providers receive some education In-depth dedicated team training	Feasibility: balance provider capacity with need for base knowledge across all providers
Provider resources (effectiveness, reach, adoption, implementation [acceptability, appropriateness])			
Written	Evidence-based clinical decision-making tools Referral guides or work-up flow diagram	Evaluation or referral flow diagram	Practical clinical resources guide decision making, address knowledge barriers, promote retention
Personnel	Development of clinical team Established specialist partnerships	Development of clinical team Establish specialist partnerships	Reduces variability in knowledge and prioritization Promotes referral and involvement of SF experts
Intervention design (effectiveness, reach, adoption, implementation [acceptability, feasibility])			
Planning	Partner involvement intervention component selection and implementation planning	Involve key partners (providers, patients, clinical leadership, RNs^e^, MAs^f^)	Design intervention to fit clinical setting Buy-in more likely if key partners involved from beginning
Iteration or scale-up or stage	Optional stage	Start in small setting and scale up over time (PDSA^g^ cycles)	Allow for adjustments to unforeseen challenges

a This table outlines key implementation topics, options, and decisions throughout the co-design process. Organized by overarching implementation components, we include options for implementation, the consensus-driven implementation plan, and the rationale for these.

b EHR: electronic health record.

c BPA: best practice alert.

d SF: sexual function.

e RN: registered nurse.

f MA: medical assistant.

g PDSA: Plan-Do-Study-Act.

With myriad potential decisions and multiple forms available for each function of the screening approach and implementation strategies, it was important to understand how decisions would impact each other. In several instances, a cascade effect in decision-making was evident in which one decision influenced and often limited options for future decisions. Throughout the iterative process, there was a need for the co-design session moderators to remind participants of the overarching considerations guiding intervention design (eg, patient privacy and comfort) and the implications of decisions made in earlier sessions as the process progressed. Below, the decision points and process for selecting intervention components and implementation strategies are described, concluding with identified strategies used by the moderator and co-moderator in facilitating discussion and consensus-building in co-design sessions.

### Intervention Design

#### Overview

Several intervention design decisions were reached easily during the co-design sessions, including the target population, how and when to introduce SD screening to patients, timing and frequency of screening, and how to tailor SD screening to individual patients, allowing for patient choice and flexibility in unique situations (Table 3). However, other decisions required a more iterative approach and, at times, discussions over multiple sessions. These decisions, and how the research team facilitated the co-design process, are described below.

#### Screening Modality and Workflow

##### Discussion Summary

Much of the discussion on screening modality (eg, written vs verbal, electronic vs paper) centered on key considerations of patient privacy and comfort. Participants considered the possibility of multiple delivery modalities or allowing patients to choose their preferred modality. These decisions were recognized as highly dependent on and interrelated with other decisions such as timing around visits and the location of SD screening. For example, if participants agreed that screening should take place at home prior to a clinic visit, that would limit screening modality options to a survey administered through the patient portal or sent to a patient’s email. Additionally, discussions around screening modality were inherently integrated with considerations related to privacy, tracking screening responses over time, and the ability to easily share results with specialists and consultants. To support discussion and decision-making, the moderator gathered additional information about the EHR patient portal between co-design sessions, particularly related to patient privacy concerns. After learning that the EHR patient portal used in their health system was incapable of restricting questionnaires to patients (vs their guardians), participants determined that screening through the EHR patient portal was not an acceptable approach.

After further integrated discussions about modality, timing, and location of screening, participants reached a consensus decision that prioritized privacy, patient comfort, and screening results storage over workflow. Participants believed the best approach was to use an EHR feature for private in-clinic screening once a patient checked in for an appointment. To accommodate this approach, participants identified a need for a new workflow in which adolescent and young adult patients would be brought into the exam room alone by the MA to allow for privacy during screening completion, with guardians brought back afterward. Beyond SD screening, participants believed that independent rooming of adolescent and young adult patients would be beneficial to optimize their health care and promote health independence.

##### Strategies to Facilitate Decision-Making

To reach consensus on screening modality and workflow, the research team collected and provided additional information to support decision-making (eg, on EHR capabilities as described above), reminded participants of key considerations identified in the discover phase (ie, privacy, comfort, workflow), and redirected conversations and preliminary decisions when in conflict with prior research findings. For example, in an early co-design session, participants identified the clinic waiting room as a potential location for screening; however, this directly conflicted with numerous patients’ explicit recommendations that screening should *not* occur in the waiting room. Reminding provider participants of these findings resulted in successful redirection.

### Management of Screening Results

#### Discussion Summary

Determination of which provider or providers would be responsible for following up on SD screening results, including speaking with the patient and developing a clinical management plan, was discussed extensively over 2 sessions prior to arriving at a consensus decision. In the discover phase, provider interviews yielded variable recommendations for who should manage SD screening results [9]: the provider seeing the patient on the day of screening, the patient’s primary oncology team, a separate dedicated SD person or team, an external specialist, or a dedicated person within the patient’s oncology team (Table 3). Initially, there were mixed opinions across the co-design group on the best approach. Participants were reminded of prior findings including the importance of patient-provider rapport and provider expertise, as well as previously identified barriers such as variation in the relative importance or priority of SD screening between providers and limited time or capacity. Participants were also re-oriented to principles of standardization and sustainability when considering the best approach. After initial discussions, participants determined that each clinical oncology team (ie, liquid, solid, neuro-oncology) should have a designated individual responsible for following up on results. One participant, who had assumed a leadership role within group discussions, expressed concerns about the feasibility of a single provider being responsible for SD management. Sharing these concerns, the research team gathered additional data regarding the estimated number of screening events per team per year. This additional information served to educate and redirect the working group, who agreed that this would be too burdensome for an individual provider and that each subspecialty team should have a sexual health team of 3 to 4 individuals instead. Once consensus was reached, participants were encouraged to consider any challenges related to rapport, workflow, and scheduling. Following discussion of these challenges, participants agreed that each sexual health team should be made up of the providers who are most often available in the clinic (eg, nurses, advanced practice providers).

#### Strategies to Facilitate Decision-Making

To reach consensus on SD screening result management, the research team gathered additional information (eg, estimated number of screening events per clinic) to support providers in reaching an informed consensus. Research team members provided guidance on decision practicality; re-oriented participants about prior study findings (patient preference for rapport); and reminded participants to consider the goals of feasibility, scalability, and sustainability in reaching their decisions.

### Implementation Strategy Selection

In addition to discussing the specifics of SD screening delivery and clinical management, the co-design participants considered implementation strategies aimed at optimizing provider buy-in, reach, adoption, scalability, and sustainability. The moderator and co-moderator introduced these co-design session topics by integrating prior qualitative interview results with information about established implementation strategies [39].

#### Reminders and Cues

Co-design participants discussed several implementation strategies to support standardization and, subsequently, scalability, reach, and adoption. Strategies included the use of automated EHR–based reminders and the modification of existing paper-based patient care documents (such as chemotherapy “roadmaps”) (Table 4) [9]. Participants determined that the latter approach, which would rely on additional effort by individual members of the medical team, was not feasible or sustainable. Participants agreed that relying on technology-based reminders would be ideal but were uncertain about what EHR-based options were available and were concerned about reminder or pop-up fatigue. After receiving more information about EHR capabilities, participants determined that building EHR-based rules for triggering the questionnaire as “due” was ideal.

Based on both established implementation science strategies and suggestions mentioned in provider interviews, the research team also initiated a discussion about the use of “audit and feedback” or tracking benchmarks for individual provider or team success. Participants were not enthusiastic about the development of benchmarks for implementation success, nor the use of audit and feedback to monitor the use of the SD screening approach with patients.

#### Education and Training

##### Discussion Summary

As in our discover phase interviews, participants acknowledged the importance of provider buy-in for implementation success. They agreed that increasing provider knowledge about SD and its importance for the pediatric oncology population, increasing provider knowledge of the screening intervention, and increasing provider self-efficacy in managing SD would facilitate provider buy-in. One key recommendation from the co-design participants was that while all providers should receive education and training, different types and depths of education and training should be developed for different individuals to align with their roles and intervention involvement (eg, MAs, sexual health team members, and nurses and providers taking care of the patient but not directly involved in SD screening or follow-up). Participants recommended varied training formats, including in-person training with role-play, online training, and easily accessible written resources.

##### Strategies to Facilitate Selection of Implementation Strategies

To facilitate decision-making regarding implementation strategies, the research team integrated prior findings into co-design sessions to support and supplement participant-brainstormed approaches. Additional information was gathered between sessions (EHR capabilities) and relayed back to the working group to clarify and confirm potential implementation options.

### Process Mapping Session

**Figure 1.**
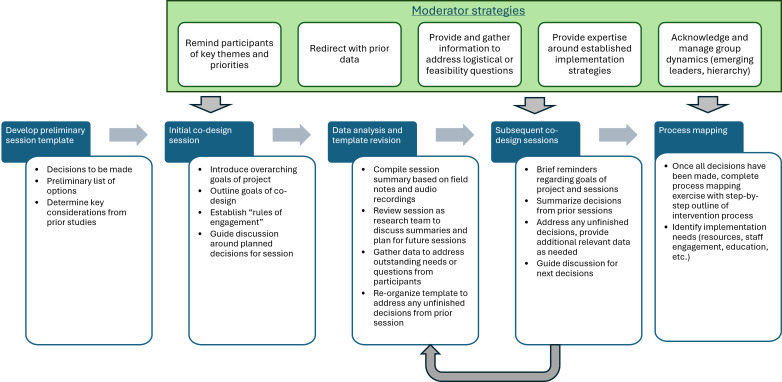
Co-design process for development of the sexual dysfunction screening intervention and implementation approach. To develop the sexual dysfunction screening approach, iterative co-design steps were completed over multiple sessions, culminating in a process mapping session. Moderator strategies were used to facilitate consensus-building and the development of a feasible, appropriate, and acceptable intervention prototype.

The primary focus of this session was a process mapping exercise outlining consensus-driven decisions from the intervention start (patient education about SD and screening approach) to completion (management of identified SD needs). In the preparation for this session, the research team compiled all decisions made in prior sessions into a process diagram (Figure 1), used to guide the discussion of every aspect of the SD screening approach.

This process helped to identify additional issues, such as potential variability in patient education at the time of diagnosis and the importance of delaying the first screening so as not to screen patients on their first visit to the oncology clinic (Table 3). Additional potentially helpful implementation strategies were identified, such as the integration of a notification system, for the clinical team upon screening completion and the development of note templates for uniform documentation of sexual health team discussions.

## Discussion

Through the engagement of provider partners, we developed a user-informed SD screening intervention and implementation prototype for adolescent and young adult childhood cancer survivors. This prototype specifically addresses previously identified barriers to successful SD screening (eg, patient or provider comfort) and prioritizes patients’ preferences and needs (eg, patient privacy). Figure 1 demonstrates the final co-design process, which was built on user-centered design methods with the goal of developing a preliminarily acceptable, feasible, scalable, and sustainable standardized SD screening approach. This process evolved, becoming more iterative and dynamic to meet participants’ needs for consensus-building. This study describes our iterative co-design method comprising the design phase of the over-arching discover-design-build-test process, which encouraged end users (providers) to integrate our prior discovery data with their own experiences, opinions, and clinical expertise and collaborate in a consensus-driven approach [16]. Consensus decisions were tailored to the context with the goal of improved alignment of needs, preferences, and workflows. This study highlights challenging decisions and co-design moderator strategies for guiding the co-design participants to consensus, providing a case study of this process. Importantly, the decisions, or “forms” of the intervention, made by participants are specific to the unique clinic setting but will remain flexible when the overall intervention design is adapted for implementation in other contexts [22,23].

Our user-centered design approach shares many characteristics with other collaborative dissemination and implementation science methods, including the use of focus groups, nominal group technique, and the Delphi method [28,29,40-44]. Each of these methods, along with user-centered design, offers a structured approach to idea generation, problem solving, and decision-making [45]. For this study, the user-centered design approach allowed us to iteratively adapt an SD screening intervention in collaboration with end users who will be responsible for or involved in intervention delivery. With the participation of providers possessing actionable insights regarding the patient population being served and the specifics of clinic workflow, co-design sessions aimed to develop an acceptable, feasible, scalable, and sustainable standardized prototype.

The importance of using an interactive iterative process was emphasized by our experiences in several of the co-design discussions wherein a decision regarding a particular intervention component or implementation strategy was later changed based on a subsequent decision. In this dynamic process, considering the full SD screening approach as a system rather than as a set of independent components was essential. While more structured methods, such as the nominal group technique and the Delphi method, may provide more assurance of equal input between participants, our process allowed for interactive and dynamic collaboration [46]. The co-design process and consensus-building aimed to achieve a middle ground among participants where decisions were collective and found to be satisfactory; this middle ground often meant that some identified needs were prioritized above others. While participants often made efforts to prioritize previously established design considerations (eg, privacy, comfort, workflow), at times the research team had to remind the group of their importance. Ultimately, the final prototype did affect workflow by requiring the development of a new patient rooming approach; this was discussed extensively over multiple sessions prior to arriving at this decision, with the goal of optimizing patient privacy, comfort, and sustainability while also promoting patient independence and self-advocacy.

Lazo-Porras et al [47] describe challenges associated with co-creation, including the need for the research team to obtain skills in facilitation and the risk of participants generating ideas that are not feasible, too specific, or out of scope. Through the conduct of our co-design project, we identified several strategies that may be used to facilitate discussion, consensus-building, and feasibility [47]. Specifically, the research team used strategies including (1) reminding participants of prior key research findings and key functions of the intervention; (2) redirecting when decisions were in conflict with these or with prior decisions; (3) gathering of feasibility data and answers to logistical questions; (4) providing information to support decisions, particularly related to internal or external policies or laws; and (5) and ensuring all participant voices were heard and valued across clinic roles [48]. Lazo-Porras et al [47] also identified the importance of careful selection of participants with diverse views, which we attempted to achieve by recruiting across roles and teams, and the use of an advisory board, which was beyond the scope of this work.

Additionally, group dynamics played a key role in the co-design process [48]. The research team noted that while all participants agreed on the importance of the intervention, several participants were particularly motivated to advocate for the intervention and think pragmatically to develop a feasible and sustainable approach. These participants emerged as informal group leaders and often served as “voices of reason” when considering various design forms. Importantly, these informal leaders did not follow the traditional medical professional hierarchy. Serving in a different role, another participant regularly identified subset populations for which the SD screening approach might be more difficult (eg, cognitively impaired, non-English reading), encouraging the group to consider these important scenarios. At times, the participants who emerged as informal “leaders” intervened to encourage the group to prioritize developing an SD screening approach that would work for “most” patients and situations. Notably, group formats have the risk of allowing for uneven participation and representation across roles, but this was not observed by the research team. Finally, there were times when the group did not reach consensus; in these scenarios, participants naturally defaulted to a “majority rules” approach for decision-making.

There are several limitations to our co-design approach. First, our participant demographics were not diverse; only female providers agreed to participate, and nearly all were White. It is possible that male and female participants may have different levels of comfort with discussing SD with adolescent and young adults, particularly of the opposite sex; this could impact participant recommendations for implementing SD screening. In the discover phase qualitative interviews, a few participants suggested attempting to match patient-provider sex for this reason [9,11]. During process mapping, some participants agreed that this would be ideal but challenging to implement. Second, our group lacked MAs, none of whom expressed interest in participating. Because MAs often serve a key role in administering patient questionnaires (such as those included in the developed prototype intervention), their involvement will be imperative as the prototype is implemented, tested, and revised. Third, our recruitment focused on frontline medical team members; involvement of clinical leaders may have resulted in additional insights about potential barriers and may have secured buy-in from this key group to facilitate future implementation [47,49]. Engagement of multilevel stakeholders and clinical leaders is recommended in implementation planning [22,38], and next steps in our research program will require engagement of clinical leaders who may feel ownership of clinic practices [50]. Fourth, our process engaged only 6 participants. While this sample size is small, we found it to be appropriate given that our methodology relied on robust participant engagement and consensus building over multiple co-design sessions, processes that are less feasible with larger groups [51]. Importantly, significant variability in the conduct and reporting of co-design processes [30-32,34] has hindered the identification of ideal sample sizes, with some engaging as few as 3 providers; as our program of research continues, the results of our co-designed intervention and implementation strategies will provide additional data to inform this decision. Finally, co-creation team composition was likely affected by self-selection bias, with those who declined to participate possibly being less enthusiastic or more skeptical about the use of standardized SD screening. Their input will be critical in the upcoming build and test phases.

 Despite these limitations, the use of iterative co-design groups enabled the collaborative development of a preliminary SD screening approach for adolescent and young adult childhood cancer survivors. This approach not only used providers’ expertise but also integrated prior data from patients and providers to inform design decisions. Judicious use of moderation techniques ensured that patient preferences, feasibility, scalability, and sustainability remained front of mind as providers reached decisions about the screening intervention components and selected key implementation strategies to finalize the screening approach prototype. In the next phase of research, the prototype SD screening approach will undergo multistep testing and adaptation in small cycles. These real-world cycles are expected to identify unforeseen barriers and inform refinements to the prototype in preparation for effectiveness testing and the eventual dissemination of the intervention.

## Abbreviations

CFIR: Consolidated Framework for Implementation Research
EHR: electronic health record
MA: medical assistant
RE-AIM: Reach, Effectiveness, Adoption, Implementation, and Maintenance
SD: sexual dysfunction
SexFS: Brief Sexual Function and Satisfaction v2.0 tool
StaRI: Standards for Reporting Implementation Studies
